# On Cold Water in Gout

**Published:** 1804-02-01

**Authors:** 


### *To the Editors of the Medical and Physical Journal.*

GENTLEMEN,

A Temporary absence from home prevented me from seeing the Number of your excellent Journal for December last, till it was too late to expect that any thing then forwarded could be inserted, else the following statement should have reached you earlier.

I acknowledge myself flattered by the honour Dr. Kinglake has conferred on me by inserting the whole of the observations on cold water a second time. It must needs be a good story that will bear being twice told. The anecdote of the late Dr. Gregory, I had been in the habit of hearing so frequently repeated, even from my earliest years, by a person entitled to my utmost confidence, and one who was an intimate friend and a warm admirer of the liberal mind and manly virtues of the Doctor, that I never even doubted of its truth. Even the Editors of the Annals of Medicine allow, that such a report was prevalent in Edinburgh at the time of the Doctor's death, and which has not previously, to my knowledge, been contradicted. Dr. Kinglake's triumph does not seem very complete, even admitting the falsehood of the statement. Nothing more than a negative proof in support of his doctrine can be derived from it, as it only shews, that persons of a gouty diathesis are liable to sudden death, even tho' they do not plunge their extremities in cold water.

One reason of my addressing you at present, Gentlemen, is to express my regret at having been the cause of bringing the respected name of the late Dr. Gregory into question on so trivial an occasion; or of occasioning to the present, my much respected Master, the trouble of contradicting the statement. My error was truly unintentional; and I trust Dr. G. and his friends will consider this acknowledgment of it, as an adequate apology.

In considering me as hostile to medical improvement, Dr. Kinglake appears evidently to mistake the tendency of my observations. No man, who has taken upon himself the grave and important duty of superintending the conduct of his fellow creatures, during the melancholy visitations of bodily disease, could, I trust, be so deficient in the common duties of humanity, as to neglect or despise any species of information that would tend to mitigate pain, or shorten the duration of misery. No fault was imputed to Dr. K. for attempting to cure the gout by the application of cold water. The effects of temperature in modifying diseased action, at present attracts much of the attention of the medical world; and when they have been more accurately determined by time and experience, will in all probability extend the dominion of regular science over the functions of the living body.

To extenuate the spirit of levity and wrangling which Dr. K. has discovered in the trifling article alluded to, I shall beg his leave to use the apology employed by Horace on a similar occasion.

> O! imitatores, servum pecus, ut mihi sæpe

> *Bilem*, sæpe *jocum* vestri movere tumultus.

What, in truth, moved my bile, was the pompous manner in which Dr. K. announced the application of cold water in gout as a novel practice, originating with himself, and for which he seemed hastily to solicit the applause due to a public benefactor. A little irony appeared not ill calculated to repel such an arrogant and unfounded assumption; but the Doctor, it seems, does not like a joke.

> Ludus enim genuit trepidum certamen, et iram:

> Ira, truces inimicitias, et funebre bellum.

With your permission then, Gentlemen, I will proceed to an investigation more serious, and more becoming professional gravity and decorum, concerning the validity of the Doctor's claim to originality in the application of cold water to the cure of the gout.

To begin at the beginning, that is, with Hippocrates, Aphorism 25, sect. 5, (I need not trouble you with the Greek text) are the following words. "Tumores autem in articulis, et dolores alisque ulcere, et *podagricos*, et convulsiones horum plurima, *frigida multa affusa et levat, et attenuat, et dolorem solvit.* Torpor enim modicus doloris solvendi vim habet. The same doctrine is repeated in nearly similar words in the chapter De humidorum usu.

Among the remedies recommended by Celsus, "De manuum et pedum articulorumque vitiis," we read as follows. "Si vero tumor calorque est, utilioraque sunt *refrigerantia, in aqua quam frigidissima articuli continentur.*" Again, "Levat spongia imposita quæ subinde ex oleo, vel aceto, *vel aqua frigidissima* exprimitur."

But, to come nearer to our own times, did the Doctor, in the pursuit of his cold water lucubrations, never stumble on a work by Hermanus Van der Heylen, entitled

Aquæ frigidæ inter inauditas et incredibiles alias effectus podagræ dolores vel sistentis, vel mirabiliter demulcentis, et ischiadicas dolores penitus extirpantis, &c." From an English translation of this book, now lying before me, I shall treat the Doctor with a quotation.

"After shewing very clearly, that pains of the gout are caused either by a hot humour, or by an acrimonious or salt one, proceeding from the liver, Vander exclaims,—"But seeing it is confessed and assented to by all physicians, *that contraries are cured by contraries*, why may I not lift up my voice, and make use of my pen, in the just praises of this our cold water?"——Vander also lays claim to the discovery of the use of cold water, tho' I do not believe he was ignorant of its having been recommended both by Hippocrates and Celsus, whatever Dr. K. may be. "But suppose," says he, "that the excellent virtues of cold water in the cure of this disease were never before discovered to the world; or that other its excellencies, in other the like cases, have hitherto been neither written nor heard of:" Suppose this, I say, must the vast abyss of physical knowledge, and the large stock of the wayes of *cures* have before this been necessarily exhausted and drawn dry? Is it impossible to say or write any thing that may be deduced out of the very principles of Nature, which may be of good use in curing of the diseases men are subject unto, and particularly of the intolerable pains of the gout and the like? Certainly there are divers, that having by experience found the excellent virtue of cold water for assuaging of those horrid torments of the gout, (which are a second hell) will be ready with a very grateful remembrance, publicly to extol the same." *Van* clearly deserves the palm for modesty; he only insinuates that he would wish to be considered as the inventor of the cold water system, but does not absolutely say he is so.

Smith's Curiosities of Common Water, a well known work, which has passed through many editions, contains abundance of recommendations in favour of the use of cold water in gout and rheumatism.

But putting aside those old fashioned books, has Dr. K. never seen the modern and truly excellent Treatise of Mr. Rigby on Animal Heat. In this work the local application of cold water in the gout, and in various exanthematous complaints, is treated of in a scientific manner, and illustrated by apposite cases and legitimate deductions.

In the sixth volume of the Medical Observations, some instances of the utility of cold in the gout, accompanied with with many pertinent reflections by the person who was himself the subject of the experiments, may be found.

And now, Gentlemen, I will leave it with you to determine, whether any man, on the revival of a practice, which has been so frequently brought forward and again abandoned, (on good grounds or not is foreign to the present argument) be entitled to call out *eureka,* as if he were the first who had passed the pons asinorum.

Whatever may be the relative merits of the three remedies, the pump, the tincture, and the cold water, it must be allowed that there was a singular coincidence in their being ushered into public notice much about the same time. Nor do I think it shewed any very reprehensible degree of timidity in a man, whose daily bread depends on his assisting poor human nature to contend against the gout, and the other various ills that flesh is heir to, to express some degree of alarm at the combined appearance of three remedies, each in its single unaided strength promising to extirpate a complaint, whose existence has in all ages contributed so liberally to the support of every branch of the faculty, and the more so, as it commonly singles out its victims among the rich and the timid. Those fears have now indeed, in great measure subsided. Of the pump not much is heard. The tincture, though if uncontradicted rumour spoke true, supported on the wings of a genius,

> Vitreo daturus

> Nomina ponto,

Is gone to the tomb of all the Capulets, and is now less heard of, and probably less employed, than Dr. Solomon's anti-impetigines. And though the celebrated Hoffman wrote a Treatise de Aqua Medicina Universali, the ravages of disease have not yet ceased to limit the numbers of mankind, nor do I believe that gout is exterminated from the face of the earth.

Conscious of having trespassed too long on your patience, I now take a final leave of Dr. Kinglake, impressed with a well founded confidence that he will, in future, entertain that kind of friendship and regard for me, which a man generally entertains for another to whom he feels himself under an obligation. Dr. K. through the medium of the Medical and Physical Journal, called for materials to assist him in making a book. I have furnished him with some, and pointed out the pregnant sources of others. The quotations I have inserted, require no other authority than a reference to the books from which they are extracted; tracted; I shall therefore still request your permission to remain shrouded in my veil of "anonymous concealment," as Dr. K. very neatly phrases it. This determination is perhaps less the result of fear of my antagonist, than of a peculiar aversion to medical controversy. Though I will not pledge myself, that when the Doctor shall have published the book with which he has threatened the world, if I do not think his facts well established, and his deductions logical, I may not start up, propria persona, and endeavour, through the aperture of a goose's quill, to throw cold water on the whole. Till that auspicious æra arrive, I shall continue to regulate my practice in the gout as I have hitherto endeavoured to do, by the precepts founded on painful personal observation of the honest and judicious Sydenham, I am, &c.

A CONSTANT READER.

To the EDITORS of the MEDICAL and PHYSICAL JOURNAL.

Gentlemen,

